# Genome-Wide Identification of Genomic Regions Associated with Body Weight and Morphometric Traits in Awassi Sheep

**DOI:** 10.3390/ani16060867

**Published:** 2026-03-10

**Authors:** Mervan Bayraktar, Hussein F. Hasan, Omer Shoshin

**Affiliations:** 1 Department of Animal Science, Faculty of Agriculture, Çukurova University, Adana 01330, Turkey; 2 National University of Science and Technology, Nasiriyah 64001, Dhi Qar, Iraq; hussein.f.hasan@nust.edu.iq; 3 Department of Physiology, College of Veterinary, Kirkuk University, Kirkuk 36001, Kirkuk, Iraq; omersh@uokirkuk.edu.iq; 4 Institute of Health Sciences, Ankara University, Ankara 06100, Turkey

**Keywords:** Awassi sheep, growth traits, multi-locus GWAS, BLUPmrMLM, candidate genes

## Abstract

In this study, we investigated the genomic basis of key body measurement and weight traits in Iraqi Awassi sheep using a multi-locus genome-wide association approach. A total of 315 yearling animals were phenotyped for body length, chest depth, heart girth, withers height, and body weight, and genotyped using the Ovine 50K SNP BeadChip. Genome-wide association analyses were performed within the BLUPmrMLM framework to improve the detection of loci with moderate-to-small effects. Significant associations were identified using an LOD-based threshold (LOD ≥ 5), followed by positional annotation of nearby genes and functional enrichment analyses to infer their potential biological relevance. Multiple genomic regions were associated with the evaluated traits. Among the most biologically plausible candidate genes were *DST* and *CFAP299* for body length, *ADAMTS8* for chest depth, *ZFPM1* and *OST4* for heart girth, *CPEB2* for body weight, and *ITGBL1*, *RBMS3*, and *THSD7B* for withers height. Functional enrichment analyses indicated the involvement of pathways related to integrin-mediated signaling, focal adhesion and integrin complexes, extracellular matrix organization, and post-transcriptional regulation, suggesting coordinated effects of cell–matrix interactions and gene-expression regulation on body size and conformation. Overall, these findings refine the genomic landscape underlying body weight and morphometric variation in Awassi sheep and provide a focused set of loci for future validation and possible application in marker-assisted and genomic selection programs.

## Simple Summary

Improving growth and body shape is an important goal in Awassi sheep because heavier and better-conformed animals can increase meat production and farm income. In Iraqi Awassi sheep, however, the genomic regions that influence differences in growth and body measurements are not well described, which limits the use of DNA information in breeding. This study aimed to identify genomic regions associated with five key traits in 315 one-year-old Iraqi Awassi sheep: body length, chest depth, chest circumference, shoulder height, and body weight. We measured these traits in animals raised under local farm conditions and examined thousands of DNA markers across the genome. We found several genomic regions that were clearly associated with variation in these traits and highlighted nearby genes that may affect tissue growth, body structure, and cell attachment and interactions with surrounding support tissues. Overall, the results provide a clearer picture of the genetic factors underlying growth and body conformation in Iraqi Awassi sheep. These findings can support the development of practical DNA-based selection tools to help breeders choose animals with better growth potential, improving productivity and supporting more efficient and sustainable sheep production.

## 1. Introduction

Awassi sheep (*Ovis aries*) are among the most important and widely distributed fat-tailed breeds in the Near East, including Iraq, due to their adaptability to arid and semi-arid production systems and their multi-purpose value [1,2,3]. However, adaptation alone does not ensure economic profitability; therefore, improving body weight and body conformation remains a central breeding objective in Awassi populations, as these traits directly influence market value, carcass characteristics, and overall production efficiency. In practical breeding settings, simple linear body measurements are often used as convenient indicators of body size and conformation because they are informative, easy to obtain under field conditions, and require minimal equipment [4,5]. However, the phenotypic expression of growth and conformation traits is shaped by complex interactions among genetics, nutrition, management, and environment, and their inheritance is typically polygenic, with many loci contributing small-to-moderate effects. Importantly, integrating genotypic information enables the dissection of this phenotypic variation by identifying loci that contribute to trait expression and by separating genetic effects from environmental noise. Therefore, identifying genomic regions associated with these traits can support more accurate selection decisions and accelerate genetic progress [6,7,8]. Genome-wide association studies (GWAS) have become a widely used approach to dissect the genetic architecture of complex quantitative traits in livestock by testing associations between genome-wide SNPs and phenotypes [9,10]. In sheep, GWAS has been successfully applied to growth and carcass-related traits, demonstrating that measurable phenotypic variation can be linked to specific genomic regions and biologically meaningful candidate genes [11,12]. Nevertheless, classical single-locus GWAS frameworks can have limited power to detect loci with moderate-to-small effects, especially when sample sizes are constrained or when many loci with correlated effects influence the trait [13]. Moreover, stringent multiple-testing corrections in single-locus GWAS can reduce sensitivity and increase false negatives, potentially missing biologically relevant signals [14,15]. To address these limitations, multi-locus GWAS methodologies have been developed. These models jointly consider multiple markers and treat SNP effects as random or mixed, improving power and effect estimation while reducing the reliance on overly conservative significance thresholds [13,16]. The mrMLM framework and its extensions have been widely adopted as representative multi-locus approaches for improving QTN detection in complex traits [13,17]. More recently, BLUPmrMLM was proposed as a computationally efficient multi-locus method that integrates best linear unbiased prediction (BLUP) with multi-locus random-SNP-effect mixed modeling to improve runtime and detection performance in large-scale datasets [18]. Collectively, these advances provide a strong methodological foundation for mapping loci associated with body weight and morphometric traits in sheep populations, where many underlying variants are expected to have modest effects. Although Awassi sheep are economically important and well adapted to regional production systems, the genomic basis of body weight and body conformation traits in Iraqi Awassi populations remains insufficiently understood. Expanding this knowledge may facilitate the development of marker-assisted strategies and, ultimately, genomic selection approaches tailored to local breeding objectives and environmental conditions. Therefore, the present study aimed to identify genomic regions associated with key body weight and morphometric traits in Iraqi Awassi sheep using a multi-locus GWAS approach.

## 2. Materials and Methods

### 2.1. Animals, Management, and Phenotypes

**Figure 1:**
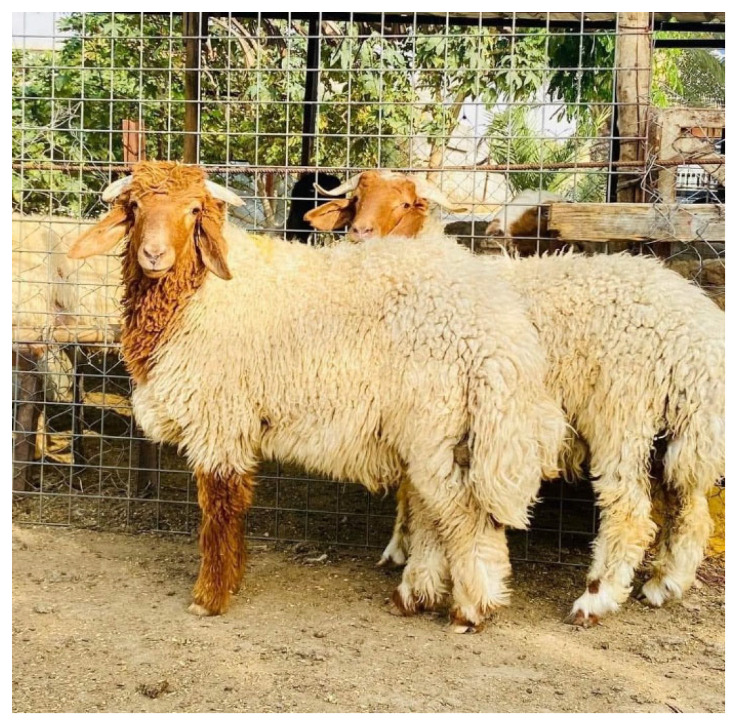
Sheep breed samples.

A total of 315 one-year-old Awassi sheep were included in this study (180 males and 135 females) (Figure 1). Animals were sampled from two flocks located in different localities within the Kirkuk region, Iraq, and were maintained under routine local production conditions. Flock (herd) of origin was recorded for each animal at sampling, as flock-based sampling may introduce subtle genetic structure through shared ancestry and local breeding practices (e.g., use of a limited number of breeding rams and non-random mating within herds). Morphometric measurements and body weight were recorded for all 315 animals, and all phenotyped individuals were subsequently genotyped using the Ovine 50K SNP BeadChip and included in downstream association analyses. Growth and body conformation traits were recorded using standardized zootechnical procedures, following FAO guidelines for phenotypic characterization of sheep and goats and the FAO measurement definitions for live-animal morphometrics [19,20]. The phenotypes included body length, chest depth, heart girth, withers height, and body weight. Linear measurements (cm) were taken with animals standing squarely on a flat surface using consistent anatomical landmarks. Heart girth was measured using a flexible measuring tape, whereas body length, chest depth, and withers height were recorded using a measuring stick. The same trained operator collected all measurements to minimize observer-related error. Body weight (kg) was measured using a calibrated livestock scale. All procedures involving animals were approved by the relevant ethics committee of Kirkuk Veterinary Teaching Hospital on 05-01-2026 (Approval No.KIK-2544S-2026) and conducted in accordance with applicable animal welfare guidelines.

### 2.2. DNA Sampling, Extraction, and SNP Genotyping

Peripheral blood (10 mL) was collected from the jugular vein of each animal into sterile vacuum tubes by trained personnel under routine veterinary practice. Only ~200 µL of whole blood was used for genomic DNA extraction. At the same time, the remaining sample was aliquoted and stored in a biobank for future genetic and molecular studies (including validation work and MSc/PhD student projects), thereby avoiding repeated resampling. Genomic DNA was extracted using the QIAamp DNA Blood Mini Kit according to the manufacturer’s instructions (Qiagen; Qiagen GmbH, Hilden, Germany; Cat. No. 51104). DNA concentration and purity were quantified by UV spectrophotometry (NanoDrop; Thermo Fisher Scientific, Waltham, MA, USA) based on absorbance at 260 and 280 nm, and samples with A260/A280 ratios between 1.80 and 1.95 were considered acceptable for genotyping. All animals were genotyped using the Illumina Ovine 50K SNP BeadChip (San Diego, CA, USA) comprising 54,241 markers distributed across the ovine genome. Genotype calling and primary data processing were performed using the manufacturer’s standard array workflow, and genotype files were exported for downstream quality control and genome-wide association analyses.

### 2.3. Genotype Quality Control

Genotype quality control (QC) was applied at both the individual and marker levels prior to GWAS using PLINK v1.9 (beta) [21]. Individuals with genotype missingness >10% were removed (--mind 0.10). SNPs were filtered by call rate and allele frequency, excluding markers with missingness > 10% (--geno 0.10) and MAF < 0.01 (--maf 0.01). Deviations from Hardy–Weinberg equilibrium were assessed, and SNPs with HWE *p* < 1 × 10^−6^ were excluded (--hwe 1 × 10^−6^). The resulting QC-passed dataset was used for downstream association analyses.

### 2.4. Principal Component Analysis (PCA)

To describe population structure and assess stratification, PCA was performed in R (v4.5.2) using the SNPRelate/gdsfmt framework. Briefly, QC-passed PLINK files were converted to GDS format, and PCA was computed using the SNPRelate function snpgdsPCA with LD pruning applied before PCA. The first two principal components (PC1–PC2) were visualized using ggplot2, and individuals were screened for outliers; PC1 and PC2 were subsequently used as covariates to account for population structure in the GWAS, where applicable.

### 2.5. Genome-Wide Association Analysis

Genome-wide association analyses were performed for each trait using the BLUPmrMLM method implemented in the mrMLM R package [17]. BLUPmrMLM is a multi-locus mixed-model GWAS strategy that improves the detection of loci with moderate-to-small effects by iteratively selecting and jointly fitting multiple markers while controlling background polygenic variation [18]. All analyses were conducted using the quality-controlled genotype dataset. Because the animals were sampled from two flocks (herds), and because flock-level management and family structure can introduce stratification, we explicitly accounted for this sampling design in the association model. Specifically, the flock (herd) of origin was included as a categorical fixed effect in the model. In addition, potential confounding due to population structure was controlled by including the first two principal components (PC1 and PC2) as covariates. At the same time, genome-wide relatedness was accounted for through a random polygenic effect based on the genomic relationship (kinship) matrix, consistent with unified mixed-model GWAS principles [22].

Within the BLUPmrMLM framework, the phenotypic vector was modeled as:

*y* = *Xβ* + *Wα* + *g* + *ε*

where y is the vector of phenotypic observations; X is the design matrix of fixed effects, including an intercept, flock (two-level factor), and covariates such as PC1 and PC2; β is the vector of fixed-effect coefficients; W is the genotype matrix (coded as 0/1/2) for SNPs included in the multi-locus step; α is the vector of SNP effects fitted by BLUPmrMLM; g is the random polygenic effect that captures genome-wide additive genetic background and relatedness among individuals; and ε is the residual error term. Random effects were assumed to follow:

g∼N(0,Kσg2),ε∼N(0,Iσe2)

where K is the genomic relationship (kinship) matrix, and I is an identity matrix (Yu et al., 2006 [22]; Li et al., 2024 [18]). This formulation controls confounding arising from both family-relatedness and between-flock structure, thereby improving the robustness of marker–trait association inference.

Association evidence was summarized using the LOD statistic provided by BLUPmrMLM, and a stringent significance threshold of LOD ≥ 5 was applied to declare significant associations [18]. For reporting and interpretation, significant SNPs were summarized at the level of independent genomic regions to avoid redundancy from clusters of nearby significant markers (likely tagging the same locus); within each region, the lead SNP was defined as the SNP with the highest LOD for the trait. Significant SNPs were subsequently consolidated into genomic regions based on physical proximity, and the lead SNP representing each region was retained for downstream gene annotation.

### 2.6. Candidate Gene Annotation

Candidate genes were identified by positional mapping around significant loci. Gene annotation was performed using Ensembl BioMart (Cambridge, UK) by retrieving genes within a ±500 kb window (1 Mb total) around each significant SNP (1 Mb total window) [23,24,25]. The ±500 kb interval was selected because the study was based on the Illumina Ovine 50K SNP BeadChip, which has an average inter-marker spacing of ~50.9 kb with non-uniform genomic coverage, so the lead SNP often represents a tag marker rather than a causal variant. Moreover, LD in sheep populations can extend over hundreds of kilobases, and empirical LD analyses indicate that useful LD typically does not extend beyond approximately 0.5 Mb, supporting a ±500 kb search interval for positional candidate gene mapping [26,27,28,29,30,31,32,33,34,35]. Overlapping windows were merged to generate non-redundant candidate gene lists for each trait, which were subsequently used for biological interpretation and enrichment analyses.

### 2.7. Functional Enrichment Analysis

Functional enrichment analyses were performed to identify Gene Ontology (GO) terms overrepresented among candidate genes identified by positional mapping ±500 kb. Enrichment was conducted using SRplot (https://www.bioinformatics.com.cn/srplot, accessed on 5 March 2026), a freely accessible web server that integrates commonly used bioinformatics analysis and visualization utilities [36]. Candidate gene lists were uploaded to SRplot and analyzed using GO over-representation analysis (ORA) against an appropriate background gene universe (all annotated genes for the corresponding species in the selected annotation database). Enrichment significance was evaluated using the ORA *p*-values and controlled for multiple comparisons using the Benjamini–Hochberg (BH) FDR procedure; GO terms with FDR-adjusted *p* < 0.05 were considered statistically significant and retained for interpretation [37].

## 3. Results

### 3.1. Descriptive Statistics

Descriptive statistics for the phenotypic traits measured in Awassi sheep are presented in Table 1 (Supplementary Materials Table S2). Body weight averaged 46.12 kg and ranged from 31.89 to 60.39 kg, with a standard deviation of 6.33 and a coefficient of variation (CV) of 13.73%. Body length had a mean of 78.93 cm (range: 62.06–91.56 cm), a standard deviation of 5.97, and a CV of 7.56%. Chest depth averaged 28.04 cm and varied between 21.22 and 35.70 cm, with a standard deviation of 2.90 and a CV of 10.34%. Heart girth had a mean of 86.97 cm (range: 71.99–105.34 cm), a standard deviation of 6.35, and a CV of 7.30%. Withers height averaged 74.62 cm and ranged from 64.45 to 86.13 cm, with a standard deviation of 4.64 and a CV of 6.22%.

### 3.2. Principal Component Analysis

**Figure 2:**
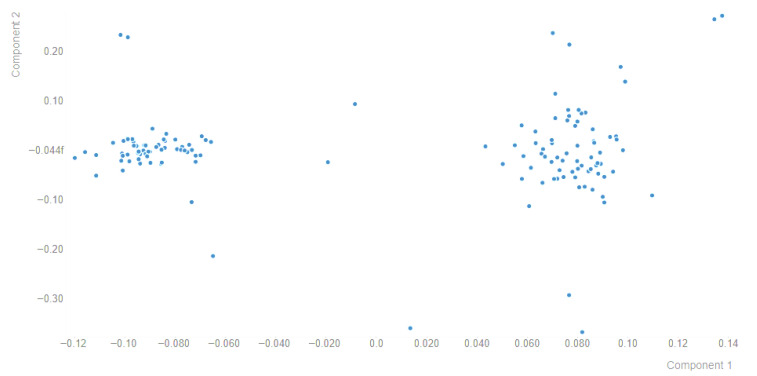
PCA plot of Iraqi Awassi sheep based on genome-wide SNP genotype data generated using the Ovine 50K SNP BeadChip. The analysis was performed to assess genomic variation and population structure among sampled animals.

Principal component analysis (PCA) was performed using genome-wide SNP genotype data. The first principal component (PC1) explained 3.19% of the total genomic variance, whereas the second principal component (PC2) explained 1.49%. Together, PC1 and PC2 accounted for 4.68% of the total variance, while the cumulative variance explained by the first three components reached 6.12%. The relatively low proportion of variance captured by the leading components suggests that the genomic variation in this population is distributed across many loci rather than being dominated by a single major axis of differentiation, indicating mild genetic stratification rather than pronounced population subdivision. The PC1–PC2 scatterplot revealed two main clusters of individuals (Figure 2). Because the animals were sampled from two flocks located in different localities within the Kirkuk region, this pattern is consistent with a modest degree of genomic differentiation related to the flock of origin. Such separation may reflect differences in allele frequencies arising from flock-specific breeding practices, partial reproductive isolation, the repeated use of a limited number of breeding rams, and within-flock relatedness. A small number of individuals were positioned farther along PC2, suggesting localized patterns of relatedness or minor genomic outliers within flocks. Overall, the PCA indicates that the sampled Awassi population is not genetically homogeneous and contains detectable but limited structure that should be considered in downstream association analyses. To reduce potential confounding caused by this genomic structure, flock was included as a fixed effect in the association models, the first two principal components were fitted as covariates, and genome-wide relatedness among individuals was accounted for through a kinship-based random polygenic effect. This combined strategy was used to improve the robustness of GWAS results by minimizing false-positive associations attributable to population structure and familial relatedness, rather than to true marker–trait relationships.

### 3.3. Genome-Wide Association Study

**Figure 3:**
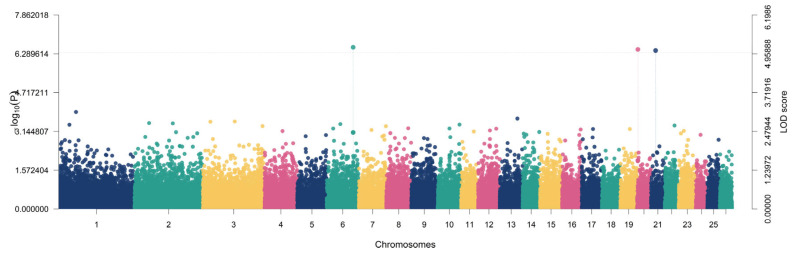
Manhattan plot showing the genome-wide association results for body length. Each point represents an SNP plotted by chromosomal position, and the y-axis indicates the LOD score. SNPs exceeding the significance threshold (LOD ≥ 5) were considered significantly associated with body length.

Genome-wide association analysis using the multi-locus BLUPmrMLM model identified significant marker–trait signals for growth and body conformation traits in Iraqi Awassi sheep (Table 2) (Supplementary Materials Table S1). BLUPmrMLM is designed to improve the detection of loci with moderate-to-small effects by fitting multiple SNP effects within a mixed-model framework. Genotyping was based on the Illumina OvineSNP50 array content (54,241 SNPs). Using the study-wide significance criterion (LOD ≥ 5), a total of 10 significant SNP–trait associations were detected across multiple autosomes, with LOD scores ranging from 5.01 to 8.39 and corresponding association strengths of −log10(*p*) = 5.81–9.29 (Table 1). For Body Length, two significant loci were detected on chromosomes 20 and 6, mapping to genes *DST* (LOD = 5.0958; −log10(*p*) = 5.8957) and *CFAP299* (LOD = 5.1655; −log10(*p*) = 5.9681), respectively. These loci explained 3.66% and 7.92% of the phenotypic variance (r^2^), with minor allele frequencies (MAF) of 0.4281 and 0.4676, respectively (Figure 3).

**Figure 4:**
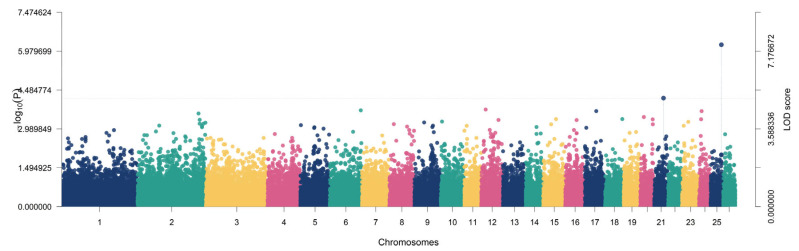
Manhattan plot showing the genome-wide association results for chest depth. Each point represents an SNP plotted by chromosomal position, and the y-axis indicates the LOD score. SNPs exceeding the significance threshold (LOD ≥ 5) were considered significantly associated with chest depth.

For chest depth, a significant association was identified on chromosome 21 near *ADAMTS8* (LOD = 5.0112; −log10(*p*) = 5.8078), explaining 4.18% of the variance with MAF = 0.3777 (Figure 4).

**Figure 5:**
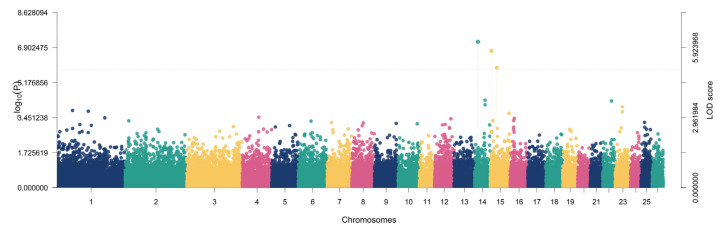
Manhattan plot showing the genome-wide association results for heart girth. Each point represents an SNP plotted by chromosomal position, and the y-axis indicates the LOD score. SNPs exceeding the significance threshold (LOD ≥ 5) were considered significantly associated with heart girth.

For heart girth, two regions exceeded the significance threshold, located on chromosomes 14 and 15, corresponding to *ZFPM1* (LOD = 6.1708; −log10(*p*) = 7.0095; r^2^ = 4.12%; MAF = 0.3597) and *OST4* (LOD = 5.7873; −log10(*p*) = 6.6130; r^2^ = 4.30%; MAF = 0.4496) (Figure 5).

**Figure 6:**
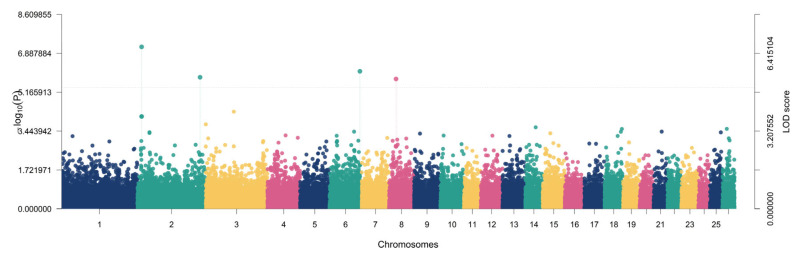
Manhattan plot showing the genome-wide association results for body weight. Each point represents an SNP plotted by chromosomal position, and the y-axis indicates the LOD score. SNPs exceeding the significance threshold (LOD ≥ 5) were considered significantly associated with body weight.

For weight, two significant signals were detected on chromosomes 2 and 6. The strongest Weight-associated signal occurred on chromosome 2 (LOD = 6.6824; −log10(*p*) = 7.5374) and explained 9.02% of phenotypic variance with a comparatively lower allele frequency (MAF = 0.2014); this locus did not map to a named gene in the reported annotation field (Table 1). A second Weight locus mapped to *CPEB2* on chromosome 6 (LOD = 5.6751; −log10(*p*) = 6.4968), explaining 3.00% of variance with MAF = 0.3705 (Figure 6).

**Figure 7:**
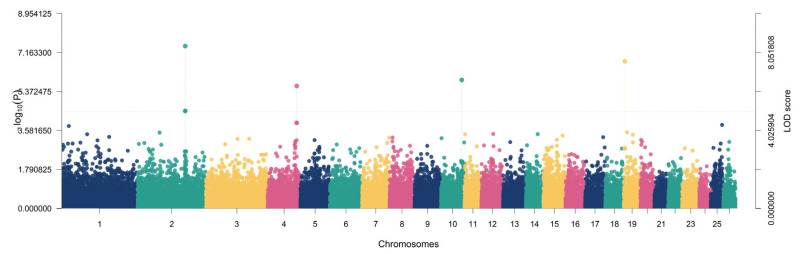
Manhattan plot showing the genome-wide association results for withers height. Each point represents an SNP plotted by chromosomal position, and the y-axis indicates the LOD score. SNPs exceeding the significance threshold (LOD ≥ 5) were considered significantly associated with withers height.

For withers height, three significant regions were identified on chromosomes 10, 19, and 2, corresponding to *ITGBL1* (LOD = 6.6352; −log10(*p*) = 7.4888; r^2^ = 6.38%; MAF = 0.4676), *RBMS3* (LOD = 7.6011; −log10(*p*) = 8.4826; r^2^ = 7.69%; MAF = 0.4712), and *THSD7B* (LOD = 8.3873; −log10(*p*) = 9.2891; r^2^ = 10.21%; MAF = 0.3957), with the *THSD7B* locus representing the strongest association signal across all traits evaluated (Figure 7).

### 3.4. Enrichment Analysis

**Figure 8:**
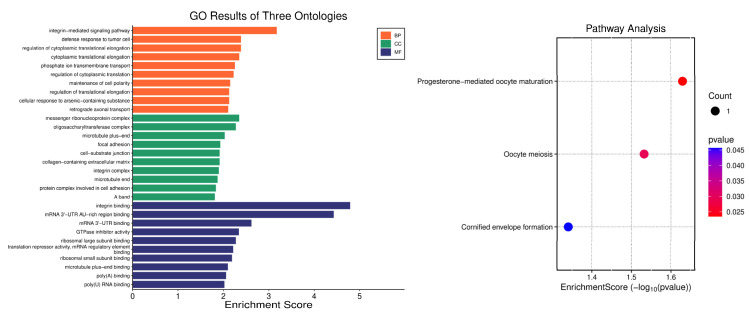
Gene ontology and pathway enrichment of the candidate gene set.

Enrichment analysis was conducted to characterize functional annotations associated with the identified gene set using Gene Ontology (GO) terms across the three ontologies (Biological Process, Cellular Component, and Molecular Function) and pathway-level enrichment (Figure 8). In the Biological Process ontology, the highest-ranking terms included integrin-mediated signaling pathway (enrichment score ≈ 3.2), defense response to tumor cell (≈2.4), regulation of cytoplasmic translational elongation (≈2.4), cytoplasmic translational elongation (≈2.4), phosphate ion transmembrane transport (≈2.3), regulation of cytoplasmic translation (≈2.2), maintenance of cell polarity (≈2.1), regulation of translational elongation (≈2.1), cellular response to arsenic-containing substance (≈2.1), and retrograde axonal transport (≈2.1). In the Cellular Component ontology, enriched categories comprised messenger ribonucleoprotein complex (≈2.3), oligosaccharyltransferase complex (≈2.2), microtubule plus-end (≈2.0), focal adhesion (≈1.9), cell–substrate junction (≈1.9), collagen-containing extracellular matrix (≈1.9), integrin complex (≈1.9), microtubule end (≈1.9), protein complex involved in cell adhesion (≈1.8), and A band (≈1.8). In the Molecular Function ontology, the top terms included integrin binding (≈4.8), mRNA 3′-UTR AU-rich region binding (≈4.4), mRNA 3′-UTR binding (≈2.6), GTPase inhibitor activity (≈2.3), ribosomal significant subunit binding (≈2.2), translation repressor activity, mRNA regulatory element binding (≈2.2), ribosomal small subunit binding (≈2.1), microtubule plus-end binding (≈2.0), poly(A) binding (≈2.0), and poly(U) RNA binding (≈2.0). Pathway enrichment identified three pathways, each supported by a single mapped gene (count = 1): Progesterone-mediated oocyte maturation (enrichment score = −log10(*p*) ≈1.62; *p* ≈ 0.025), Oocyte meiosis (≈1.52; *p* ≈ 0.030), and Cornified envelope formation (≈1.33; *p* ≈ 0.045).

## 4. Discussion

Body weight and linear body measurements are complex, polygenic traits influenced by many small-to-moderate-effect loci and by environmental factors, age, sex, nutrition, and measurement conditions. A dispersed genetic architecture for mature body size has been repeatedly reported across sheep breeds [38]. Therefore, our results should be viewed as candidate signals that require replication, fine-mapping, and functional validation [39]. Descriptive statistics show moderate within-population variation: body weight has a higher CV (~13.7%) than linear traits (~6.2–10.3%), consistent with body weight integrating skeletal size, muscling, gut fill, and fat, which are more sensitive to short-term management and environmental conditions. Body weight is typically correlated with key linear measures—especially heart girth and chest depth—and heart girth is frequently reported as a strong proxy for body weight in Awassi and multi-breed settings [40,41]. Genetically, signals for linear traits may affect weight via shared growth determinants, whereas weight-specific loci may more strongly reflect pathways related to body composition and metabolic efficiency [42]. Previous Awassi studies reporting low-to-moderate heritability for these traits indicate additive genetic variance alongside substantial environmental effects; thus, association signals are expected to explain only a small fraction of total variance, and r^2^ estimates should be interpreted cautiously [12,43].

PCA indicated that PC1 and PC2 capture only a small fraction of genome-wide variance (~3.19% and ~1.49%), with most individuals forming a tight cluster and a small subset showing more extreme positions along PC2. Low variance explained by the leading PCs is common and can still reflect meaningful stratification; the key issue is whether even subtle structure could bias association results if left unmodeled [44,45]. The PC2 outliers may indicate cryptic relatedness, introgression, or technical artifacts, which can inflate test statistics without appropriate control [46]. Accordingly, applying a mixed-model GWAS framework is methodologically justified, as it models polygenic background and relatedness, reducing confounding while maintaining power [17,18,46].

The detected SNP–trait associations explain modest fractions of phenotypic variance (≈3–10%), with the strongest signal for withers height near *THSD7B* (r^2^ ≈10.2%). Such effect sizes are consistent with a broadly polygenic architecture for body-size-related traits [38]. The results are also consistent with cross-species evidence that body size and growth traits are influenced by many genes involved in skeletal development, extracellular matrix remodeling, mechanotransduction, and growth factor signaling [42]. For body length, the mapping of a significant locus to *DST* (dystonin) provides a plausible mechanistic foothold, and mouse experiments show that *DST* isoform disruptions can produce muscle pathology, indicating an essential role in muscle integrity and cytoskeletal organization [47]. Reviews further describe *BPAG1/DST* as a plakin-family protein with multiple cytoskeleton-binding domains, consistent with a role in integrating mechanical stability across tissues [48,49]. While body length is often treated as a skeletal dimension, cytoskeletal and adhesion networks can plausibly contribute to linear dimensions via effects on muscle growth, posture, and musculoskeletal patterning [50]. The second body-length candidate, *CFAP299,* is annotated as a cilia- and flagella-associated gene and is described as required for normal motile cilia/flagella function [51,52]. Cilia biology is linked to skeletal growth through Hedgehog signaling, mechanosensation, and growth plate dynamics. Bangs and Anderson [53] synthesize extensive evidence that primary cilia are essential for mammalian Hedgehog signaling, and Moore and Jacobs) discuss the growth-plate primary cilium as a nexus integrating signals that tune longitudinal bone growth. Reviews focused on cartilage development similarly emphasize that primary cilia regulate cartilage matrix secretion, endochondral ossification, and mechanotransduction, thereby influencing skeletal dimensions [54,55]. A locus near a cilia-associated gene could reflect direct developmental pathways or linkage disequilibrium with nearby regulatory elements, but still requires validation in sheep tissues and stages. For chest depth, association near *ADAMTS8* highlights extracellular matrix remodeling as a relevant axis. Reviews describe *ADAMTS* proteins as *ECM*-associated proteases involved in matrix turnover and tissue remodeling [56], supported by mechanistic work on *ADAMTS8* activity and regulation [57], and broader syntheses linking *ADAM/ADAMTS* proteases to cell proliferation, migration, and *ECM* remodeling [58].

Heart girth is a composite measurement with a strong empirical association to body weight, and meta-analytic evidence supports its robustness as a predictor of body weight across sheep breeds [40]. The association near *ZFPM1* (*FOG1*) invites interpretation through developmental transcriptional regulation. Amigo et al. (2009) [59] describe *FOG-1* as a nuclear protein that interacts with *GATA* factors, and mechanistic studies show that *GATA–FOG* interactions can recruit chromatin remodeling complexes and regulate developmental gene expression programs [60]. Because *ZFPM1* is most prominently discussed in hematopoietic contexts, a conservative interpretation is that this locus may influence growth through pleiotropic developmental pathways or correlate with overall body size, rather than a heart-specific mechanism, and should be tested via expression profiling and replication. The heart-girth association near *OST4* underscores the involvement of protein-processing pathways. Dumax-Vorzet et al. (2013) [61] show that *OST4* assembles into native oligosaccharyltransferase complexes and that its depletion destabilizes *OST* isoforms, indicating a structural role in maintaining the stability of the N-glycosylation machinery. This is consistent with broader descriptions of the oligosaccharyltransferase complex as central to N-linked glycosylation and critical for the folding and function of secretory and membrane proteins [62]. Polymorphisms in glycosylation pathways could plausibly contribute to growth and conformation phenotypes. However, the literature more often links these pathways to developmental disorders and cellular fitness than to quantitative variation in livestock body measurements [62]. For body weight, the identification of *CPEB2* as a candidate gene is noteworthy because it connects growth phenotypes to post-transcriptional regulation of gene expression. NCBI gene curation notes roles for *CPEB2* in translational regulation in mouse spermatogenesis, consistent with its canonical identification as a cytoplasmic polyadenylation element-binding protein and regulator of mRNA translation [63]. Experimental evidence in mice indicates that *CPEB2* deficiency can alter weight gain and adiposity, with *CPEB2*-knockout mice exhibiting increased weight gain and body fat accumulation [64]. Related mechanistic literature shows that *CPEB2* can regulate translation of specific mRNAs in neuronal contexts and contribute to physiological phenotypes via translational control [64]. Although the phenotypic target differs, body weight in sheep versus adiposity/neural plasticity in mice. The convergent theme translation as a regulator of cell state and growth provides biologically coherent support for considering *CPEB2* as a credible candidate affecting energy balance, tissue growth, or developmental trajectories.

The strongest cluster of associations appears for withers height, mapping to *ITGBL1*, *RBMS3*, and *THSD7B*. Song et al. (2018) [65] provide particularly relevant animal-model evidence by showing that *ITGBL1* modulates integrin activity in developing chondrocytes and promotes chondrogenesis, directly implicating this molecule in cartilage biology. Because longitudinal skeletal growth and height traits are fundamentally shaped by growth plate chondrocyte proliferation and differentiation, an *ITGBL1* signal supports a plausible skeletal-development mechanism for withers height. This interpretation aligns with integrin biology more broadly: integrins link the extracellular matrix to intracellular cytoskeletal and signaling networks. They can transduce mechanical cues that influence anabolic and growth-related pathways [50,66].

*RBMS3* provides an independent, convergent cartilage-development signal supported by vertebrate models. Jayasena et al. (2012) [67] show in zebrafish that *RBMS3* knockdown results in craniofacial defects and that Rbms3 regulates cartilage differentiation effectors via post-transcriptional stabilization of Smad2 transcripts, linking *RBMS3* to *TGF β* receptor pathway outputs and cartilage development. This mechanism resonates with broader skeletal biology, in which *TGF-β/BMP* signaling governs chondrocyte and osteoblast differentiation, skeletal patterning, and postnatal homeostasis [68]. The concurrence of *ITGBL1* and *RBMS3* therefore suggests that withers height may be sensitive to pathways governing cartilage differentiation and mechanobiological integration.

*THSD7B* has limited direct evidence in livestock quantitative-trait studies; however, curated annotations indicate roles in actin cytoskeletal organization and membrane localization, plausibly linking it to cell adhesion and cytoskeletal remodeling [69]. Cell–*ECM* adhesion and cytoskeletal organization are connected to skeletal growth because *β1*-integrin–dependent adhesion and F-actin organization in chondrocytes are required for normal growth-plate architecture and endochondral bone formation, influencing skeletal dimensions such as withers height [38,70]. At the same time, GWAS signals may tag noncoding regulatory variants, and the nearest gene may not be causal due to LD; thus, *THSD7B* is presented as a plausible positional candidate, pending LD visualization, conditional analyses, and fine-mapping [71].

The enrichment analysis places integrin-mediated signaling, focal adhesion, cell–substrate junction, collagen-containing extracellular matrix, and integrin binding among the highest-ranking terms, while also highlighting mRNA binding and translation-related functions. Integrins and focal adhesion systems are central platforms for mechanotransduction, linking *ECM* forces to intracellular biochemical pathways that regulate protein synthesis, cellular hypertrophy, and differentiation in musculoskeletal tissues [50,66,72]. Growth plate chondrocytes and developing cartilage are also sensitive to mechanical and matrix cues, with primary cilia and adhesion signaling operating as interconnected sensing hubs in skeletal development [73,74]. 

The prominence of translation and mRNA-binding terms is consistent with the identified candidate genes. *RBMS3* is an RNA-binding protein implicated in post-transcriptional regulation in cartilage development [67], and *CPEB2* is a translational regulator with demonstrated physiological impacts in mouse models, including body weight and metabolic remodeling contexts [75]. Mechanical cues transmitted through integrins and focal adhesions can influence downstream anabolic signaling and protein synthesis, thereby shaping tissue-level growth outcomes [50].

The pathway-level enrichment results (progesterone-mediated oocyte maturation, oocyte meiosis, and cornified envelope formation) appear to be supported by single-mapped genes each. Results supported by only one gene should be interpreted as hypothesis-generating rather than confirmatory. Reimand et al. (2019) [39] emphasize that pathway enrichment requires careful attention to gene set size, background definitions, and multiple testing. In practical terms, the GO-level signals related to integrin/adhesion and translation appear more internally consistent with the candidate-gene list and the phenotypes studied than the single-gene pathway hits, and thus deserve greater interpretive weight.

The use of a multilocus mixed-model approach is an important methodological choice given the polygenic nature of growth traits. Zhang et al. (2020) [17] argue that multilocus GWAS methods can capture loci missed by single-locus approaches and provide an integrated platform for detecting multiple QTNs underlying complex traits. Li et al. (2024) [18]. At the same time, the LOD threshold adopted here (LOD ≥ 5) is more stringent than thresholds sometimes used in mrMLM contexts (often LOD ≈ 3 is discussed as a practical default in methodological descriptions), implying a conservative stance that may reduce false positives but also decrease sensitivity to small-effect loci [18]. This matters for interpretation: the detected signals may represent a subset of the strongest effects rather than an exhaustive portrait of the genetic architecture of these traits. Some candidates (*ITGBL1*, *RBMS3*, *DST*, *CPEB2*) are supported by mechanistic or developmental data in animal models linking them to chondrocyte biology, cartilage development, musculoskeletal integrity, or growth-related physiology [47,65,67,75]. Others (*THSD7B*, *OST4*, *ZFPM1*) are biologically plausible but less directly tied to quantitative musculoskeletal traits and therefore warrant cautious framing as positional candidates requiring confirmation [60,61].

## 5. Conclusions

This study provides genome-wide evidence that economically relevant growth and body conformation variation in Iraqi Awassi sheep is underpinned by a polygenic architecture in which multiple loci contribute measurable, trait-specific effects. The overall pattern of genome-wide variation is consistent with weak-to-moderate population structure, supporting the suitability of mixed-model, multi-locus association mapping for signal discovery in this dataset. Importantly, the functional context of the implicated genomic regions points to biologically coherent mechanisms related to cell–matrix interactions and adhesion signaling, cytoskeletal organization, and post-transcriptional regulation, processes that plausibly contribute to growth and morphometric development. Collectively, these findings establish an initial genomic resource for the Iraqi Awassi, offering biologically interpretable candidate regions that can be followed by replication in independent cohorts, fine-mapping, and functional genomics, and ultimately integrated into marker-assisted or genomic improvement strategies tailored to local production systems.

## Figures and Tables

**Table 1:** Descriptive statistics for the phenotypic traits evaluated.

Trait	Mean	Max	Min	SD	SE	CV (%)
BodyWeight (kg)	46.12	60.39	31.89	6.33	0.36	13.73
BodyLength (cm)	78.93	91.56	62.06	5.97	0.34	7.56
ChestDepth (cm)	28.04	35.70	21.22	2.90	0.16	10.34
HeartGirth (cm)	86.97	105.34	71.99	6.35	0.36	7.30
WithersHeight (cm)	74.62	86.13	64.45	4.64	0.26	6.22

**Table 2:** Significant SNP–trait associations detected by BLUPmrMLM in Awassi sheep.

Trait	External Gene	Chromosome	LOD Score	−log10(*p*)	r^2^ (%)	MAF
BodyLength	*DST*	20	5.0958	5.8957	3.6609	0.4281
BodyLength	*CFAP299*	6	5.1655	5.9681	7.9162	0.4676
ChestDepth	*ADAMTS8*	21	5.0112	5.8078	4.1769	0.3777
HeartGirth	*zfpm1*	14	6.1708	7.0095	4.1237	0.3597
HeartGirth	*OST4*	15	5.7873	6.613	4.3038	0.4496
Weight	*CPEB2*	6	5.6751	6.4968	2.9951	0.3705
WithersHeight	*ITGBL1*	10	6.6352	7.4888	6.381	0.4676
WithersHeight	*RBMS3*	19	7.6011	8.4826	7.6919	0.4712
WithersHeight	*THSD7B*	2	8.3873	9.2891	10.2127	0.3957

## Data Availability

The data presented in this study are available from the corresponding authors upon request.

## Supplementary Materials

The following supporting information can be downloaded at: https://www.mdpi.com/article/10.3390/ani16060867/s1, Table S1. Significant marker–trait associations in Awassi sheep. Table S2. Individual-level raw phenotypic measurements of Awassi sheep.
